# Cognitive data harmonization in the ADRC Network and beyond—Past, present, and future

**DOI:** 10.1002/alz.71086

**Published:** 2026-01-18

**Authors:** Paul K. Crane, Changye Li, Trevor Cohen, Aaron Seitz, Emma Rhodes, Seo‐Eun Choi, Michael Lee, Shubhabrata Mukherjee, Connie Nakano, Samuel Albertson, Ali Asadollahi, Karen Velderrain‐Lopez, Jeanne Gallée, Laura A. Rabin, Leslie Gaynor, Logan Dumitrescu, Shannon Turner, Timothy J. Hohman, Laura E. Gibbons, Emily H. Trittschuh, Andrew J. Saykin, Jesse Mez

**Affiliations:** ^1^ Department of Medicine University of Washington Seattle Washington USA; ^2^ Department of Biomedical Informatics University of Washington Seattle Washington USA; ^3^ Departments of Psychology Game Design, and Physical Therapy Movement and Rehabilitation Services Center for Cognitive and Brain Health Northeastern University Boston Massachusetts USA; ^4^ Department of Neurology University of Pennsylvania Philadelphia Pennsylvania USA; ^5^ Department of Psychology Brooklyn College, City University of New York Brooklyn New York USA; ^6^ Vanderbilt Memory and Alzheimer's Center Vanderbilt University Medical Center Nashville Tennessee USA; ^7^ GRECC VA Puget Sound Health Care System Seattle Washington USA; ^8^ Department of Psychiatry and Behavioral Sciences University of Washington Seattle Washington USA; ^9^ Indiana Alzheimer's Disease Research Center and Department of Radiology and Imaging Sciences Indiana University School of Medicine Indianapolis Indiana USA; ^10^ Department of Neurology Boston University Chobanian & Avedisian School of Medicine Boston Massachusetts USA

**Keywords:** cognition, digital assessment, harmonization, psychometrics, Uniform Data Set (UDS)

## Abstract

The National Alzheimer's Coordinating Center (NACC) Uniform Data Set (UDS) includes extensive cognitive data contributed by Alzheimer's Disease Research Centers (ADRCs) since 2005. The UDS has evolved over time and is on its fourth iteration. In addition to this core dataset, individual ADRCs have administered augmented neuropsychological batteries to research participants that go beyond the UDS. Here we describe ongoing efforts and future plans to optimize use of these data with modern psychometric methods. Modern psychometric methods address challenges from an evolving battery of cognitive tests. To date, most efforts using modern psychometric methods have focused on harmonization and co‐calibration of ADRCs’ UDS and non‐UDS cognitive data, while recent efforts address additional areas such as subjective cognitive impairment. Modern psychometric methods provide a workable framework for anticipated future developments, including digital cognitive testing and analyses of spoken responses. These methods facilitate optimal use of NACC's data riches to further its mission to facilitate collaborative research.

**Highlights:**

- National Alzheimer's Coordinating Center (NACC) data collection includes an extensive cognitive battery that has changed over time.
- An ambitious project has harmonized and co‐calibrated cognitive domain scores for memory, executive functioning, and language. Scores and their standard errors are available from the NACC.
- Those scores are co‐calibrated with domain scores from many additional studies, facilitating cross‐study scientific investigation.
- Future opportunities include digital data collection, consideration of neuropsychiatric symptoms and subjective cognitive impairment, and other uses of the granular cognitive data.

## 1 THE PAST: DESIGN OF THE COGNITIVE BATTERY, IMPLEMENTATION, AND DEVELOPING A CROSSWALK

Alzheimer's Disease Research Centers (ADRCs) were established by Congress in 1984 and have as their mission to be national resources for advancing research and clinical care for those with Alzheimer's disease (AD) dementia and dementia due to AD‐related disorders (ADRDs). Currently, there are 37 ADRCs across the country, all funded through the National Institute on Aging (NIA). The NIA, in 1999, also funded the National Alzheimer's Coordinating Center (NACC) as a coordinating center hub for the ADRC Program.

Neuropsychological test data were identified as critically important in the evaluation of research participants involved with ADRC studies from the very beginning. AD dementia and ADRDs are clinically defined based on measurable declines in cognition and their negative impact on functioning in daily life. Patterns of strengths and weaknesses across different cognitive domains and trajectories of change over time are seen as critically important in the evaluation and monitoring of people with these conditions.^1^

Although there was agreement that cognition was important to assess, there was no agreement on which specific neuropsychological tests to use. Initial efforts in the late 1980s at harmonization included CERAD (the Consortium to Establish a Registry for Alzheimer's Disease). An early article stated, “The specific aims of the CERAD study were to develop, standardize, and test the reliability of a brief clinical and neuropsychological assessment of AD. We designed the clinical and cognitive test batteries to characterize the primary manifestations of AD, discriminate between memory and cognitive changes associated with normal aging and those noted in mild AD, and measure the progression of dementia during the illness.” That article reported on findings from 354 people with AD dementia and 278 people without dementia.^2^ These and other efforts paved the way for the ADRCs to collect a common core group of cognitive variables and to report findings centrally to the NACC.

The initial version of the NACC Uniform Data Set (UDS) was implemented in 2005 across all ADRCs and comprised a battery of brief neuropsychological measures administered across multiple ADRCs.^3^ The UDS has been updated multiple times, resulting in four versions. A major shift was the introduction of UDS version 3 (UDS 3).^4^ In this version, tests from UDS 1 and 2 with intellectual property (IP) considerations were replaced with tests with less entangled IP considerations. Although replacement tests were selected to reflect the same cognitive constructs as their predecessors, this notable change in neuropsychological tests led to important challenges when comparing newer data to older data, especially when considering intra‐individual data over time. To address this issue, a cross‐walk study was performed where a sample of participants was administered both UDS 2 and UDS 3. With these data, equipercentile approaches were performed to obtain UDS 2 to UDS 3 test equivalences for four specific tests.^5^ A subsequent study administered only the Logical Memory and Craft stories to a diverse sample from the Einstein Aging Study and used equipercentile methods similar to those from the cross‐walk study.^6^

**RESEARCH IN CONTEXT**

1. **Systematic review**: The authors reviewed the literature using traditional (e.g., PubMed) sources. Both classical test theory and modern psychometric methods have been deployed to address harmonization challenges with National Alzheimer's Coordinating Center (NACC) cognitive data, as discussed in the article.
2. **Interpretation**: The vast riches of NACC's cognitive and neuropsychiatric data are well served by efforts to harmonize and co‐calibrate based on modern psychometric approaches. These flexible models are also well situated to address newer forms of data collection anticipated in the coming years.
3. **Future directions**: Co‐calibrated scores for cognitive data collected by the NACC are already available and can be used by investigators making use of NACC's data and specimen resources. These scores can be improved with additional data that are currently housed in each Alzheimer's Disease Research Center (ADRC) but not presently shared with the NACC. The extension of modern psychometric methods to neuropsychiatric symptoms has begun and needs further development. Extensions to address new opportunities and challenges with digital data collection are envisioned as described in the article.

## 2 THE PRESENT: INITIAL CO‐CALIBRATION OF COGNITIVE DATA FROM NACC USING MODERN PSYCHOMETRIC METHODS

Our group has taken a different approach to harmonization of cognitive data using modern psychometric methods that have been used for decades in high‐stakes educational testing settings for developing and deploying item banks.^7^, ^8^, ^9^, ^10^ For example, this approach allows for two students taking the Scholastic Aptitude Test (the SAT) who are seated at the same examination table to have test booklets with no items in common. Yet both students receive scores for reading comprehension, mathematics, and so on that are on the same metric as each other, because all of the items are calibrated together (i.e., “co‐calibrated”) in an item bank. The item banks are large, and test forms can be generated with non‐overlapping content, which makes it possible for booklets to be used at the same examination table by different students without worrying about cheating. With these approaches, standardized test scores can be compared directly even though individuals are not administered the same test items. The modern psychometric framework has proven to be very useful in the setting of educational testing for decades.

We have found these methods in general and bifactor models in particular to be useful for addressing some of the complexities in the neuropsychology space.^11^, ^12^, ^13^, ^14^ These methods avoid some of the very strong and likely violated assumptions needed for classical test theory methods, such as summing up all the points or obtaining *z*‐scores for each test and adding or averaging those together to generate a score.^15^ Examples of the assumptions made by classical test theory approaches include that all test items (e.g., category fluency and naming) measuring a given construct (e.g., language) are exchangeable and that they should be given equal weight,^15^ which does not seem plausible.

In our role as the Cognition Core for the Alzheimer's Disease Sequencing Project Phenotype Harmonization Consortium (ADSP‐PHC), we have applied modern psychometric methods to multiple data sets—including NACC—to develop harmonized, co‐calibrated scores across multiple studies. A note on these terms: *harmonized* refers to efforts to make data from two or more studies more comparable to each other, whereas *co‐calibrated* is a more stringent term that means “calibrated together” such that data are on the same metric. We have found modern psychometric approaches to be well‐suited to the task of co‐calibration. Other methods could theoretically facilitate harmonized but not co‐calibrated scores.

As part of a special issue of the journal *Neuropsychology*, we described the methodological details of our approaches for co‐calibrating cognitive test data.^16^ Several of the quality control steps we emphasized are facilitated by the extensive ADRC and NACC infrastructure. ADRCs administer the current cognitive battery and report results to NACC in a uniform way, with rigorous training and careful documentation.

In our processing and co‐calibration of these cognitive data, we consider each item in the cognitive battery from all versions of the UDS, assigning each item to a primary domain—memory, executive functioning/attention, language, visuospatial, or other—with our panel of experts, which includes two neuropsychologists (E.T. and A.J.S.) and a behavioral neurologist (J.M.). We also carefully consider whether the item is novel to our processes or whether it may have been included in a study we have encountered previously. We refer to items we have encountered as a candidate anchor or linking item. These candidate anchor items from previous studies are very important in our co‐calibration efforts, as we use previously derived item parameters for candidate anchor items that we confirm can act as anchor items. In this way, we continue to extend our item banks for each domain with each new study we harmonize and co‐calibrate.

We use structural equation modeling to determine item parameters and extract co‐calibrated cognitive scores for each domain at each study visit. We use bifactor models to account for covariance shared by items beyond that related to the cognitive domain. For example, for the memory domain, list learning tests have data from many learning trials of the same list of words. The number of words learned on each trial is correlated beyond their relationship with overall memory ability. In bifactor models, secondary factors account for this method‐related covariance.^11^, ^12^, ^13^, ^14^ Our workflows enabled us to obtain co‐calibrated scores for three cognitive domains—memory, executive functioning/attention, and language—for the entire NACC dataset, across all versions of the UDS, as outlined in our prior article.^16^

In a separate article published in this special issue of *Alzheimer's & Dementia*, we demonstrate the importance of these methods for considering longitudinal change, particularly for people whose data are collected on different UDS versions at different study visits. As we demonstrate, a substantial number of individuals had longitudinal data collected over time using more than one UDS version.^17^

Our co‐calibration efforts have been motivated, at least in part, to facilitate genetic analyses of phenotypes in the ADSP. The first phenotype evaluated by the ADSP was AD dementia versus cognitively normal elderly control case definition. There are many advantages to that phenotype—research definitions of AD dementia have been widely used in the field with criteria established in the early 1980s^18^—so the AD dementia case–control phenotype is a logical and reasonable place to start.

But the case–control design may be inefficient, as each person is included only once in the analysis as a case or as a control. There may be utility in extending beyond the case–control analyses. The ADSP‐PHC considers a variety of endophenotypes, which are continuous and intermediate analogues of the case–control phenotype, which may be related to genetic factors and thus be used to illuminate the genetic architecture of AD dementia. There are many genetic analysis success stories where the endophenotype strategy has been deployed. The intermediate endophenotypes are different from the primary phenotypes, but they have desirable statistical properties such as a continuous rather than dichotomous distribution, which enables greater statistical power from a given sample size.

Even with the endophenotype strategy, however, required sample sizes for adequately powered analyses of 3 billion base pairs of sequence data are very large, beyond the sample size available from any single study. It thus becomes necessary to consider data beyond any particular study to ensure adequate power for genetic sequence analyses.

NACC data are an important contributor to the ADSP‐PHC, which seeks to harmonize data across studies in consideration of a multitude of AD and ADRD‐related endophenotypes with the aim of facilitating these types of genetic analyses. Investigators have used NACC co‐calibrated neuropsychological test data to study associations with age, race, and apolipoprotein E (*APOE*) genotype^19^ and have considered these data alongside data from multiple other studies in important genetic analyses.^20^, ^21^, ^22^, ^23^ They have also used co‐calibrated scores from our group for non‐genetic studies, for instance, to study the dynamics of cognitive changes over time.^24^

Cognitive data can also be integrated with other types of data to generate important AD‐related phenotypes. One area of research focuses on resilience, which refers to the capacity of the brain to maintain cognition and function with aging and disease.^25^ Our co‐calibrated scores for NACC data have been used together with neuropathology and imaging data to model and study cognitive resilience.^26^, ^27^ Cognitive scores can also be used to define “super‐agers,” which are defined as older individuals who retain cognitive abilities comparable to those of individuals several decades younger. Both of these phenotypes can be examined alongside genetic data to identify protective variants.^28^ NACC cognitive data have also been analyzed together with amyloid positron emission tomography (PET) data to understand how positive amyloid biomarker status may predict future cognitive trajectories.^29^

## 3 THE FUTURE: INTEGRATING OTHER COGNITIVE DATA WITH NACC DATA

Until UDS 4, the cognitive battery was limited by the absence of a standardized test to assess verbal list learning as part of the assessment of memory, one of AD's cardinal features. At the beginning of the UDS, centers had already collected extensive longitudinal data with list learning assessments such as the CERAD assessment,^2^ the Rey Auditory Verbal Learning Test (AVLT),^30^ the California Verbal Learning Test (CVLT),^31^ or the Hopkins Verbal Learning Test (HVLT).^32^ Each ADRC was understandably reluctant to replace these tests with a different list learning assessment, given past longitudinal data collection, despite the recognized benefits of standardized data collection across ADRCs.^33^ In the UDS 4, ADRCs can choose to administer the CERAD Word List or AVLT; modern psychometric analyses as described here helped inform that decision.

In 2009, Weintraub and colleagues undertook a survey of ADRC Clinical Core leaders to better appreciate the numbers and types of additional neuropsychological tests being administered beyond the UDS at research participants’ visits.^33^ It makes sense that incorporating the non‐UDS neuropsychological data from these sites, across participants and across visits, should improve measurement precision, which can be thought of as the tightness of the estimated ability level, with the converse of measurement precision being measurement error.^34^

Furthermore, the visuospatial domain is not well assessed with the earlier UDS versions. Due to a paucity of items, we could not generate a co‐calibrated visuospatial score from UDS data alone, even though we have a visuospatial item bank and have successfully generated visuospatial scores for other studies.^16^ Incorporating individual ADRC‐specific non‐UDS data would permit visuospatial score inclusion alongside the memory, executive functioning, and language scores we generate from the UDS itself. In this special issue, Choi and colleagues illustrate this by using data from the University of Pittsburgh ADRC to demonstrate these issues. We show that adding additional data permits us to obtain visuospatial scores, and that items in other domains substantially improve measurement precision, which enhances statistical power to detect changes over time.^35^

Choi et al. demonstrate that there is considerable value in incorporating additional data from individual ADRCs from visits that have already occurred, and there may be substantial improvements in measurement precision afforded by incorporating these additional neuropsychological items from the same participant visit beyond the UDS‐required elements reported to NACC.^35^ Moving forward, UDS 4 includes two options for list learning tasks (i.e., CERAD Word List or Rey AVLT), which begin to address the past limitation. Furthermore, there have been preliminary discussions to ensure that sites can report all cognitive data collected prospectively to NACC—both UDS data and additional site‐specific data such as those analyzed in Choi et al. Future prospective efforts may enable efficient identification of non‐UDS data from study visits. Incorporating additional data, as for the University of Pittsburgh ADRC, took considerable effort on the part of both the ADRC site staff and our team. Several University of Pittsburgh ADRC team members involved in data‐collection decisions from many years ago were no longer available, so determining details like which test version in particular was administered required in‐depth investigation. Prospectively receiving all cognitive data at NACC, along with metadata close to the time of administration, will make harmonization beyond the UDS much more efficient.

## 4 THE POTENTIAL FUTURE: DIGITAL DATA CAPTURE AND ADVANCED ANALYTICS

Digital data collection for cognitive assessments presents transformative opportunities for harmonizing neuropsychological evaluations.^36^, ^37^, ^38^ One approach uses traditional tests but leverages digital administration. Carefully adapting established “paper and pencil” neuropsychological measures for digital administration can preserve critically important aspects as measured with the traditional approach while simultaneously expanding the depth and breadth of collected data. Alternatively, digital cognitive evaluations administered on computers, smartphones, or wearable devices can capture traditional scoring elements alongside a potentially rich array of behavioral measures, including response latencies,^39^ fine motor responses, interaction patterns between test administrators and participants,^40^, ^41^ and voice^42^, ^43^ and paralinguistic features^44^, ^45^ recorded during the administration of cognitive tests. Multi‐dimensional profiles captured in these ways, beyond the traditional scoring rubrics, facilitate an opportunity to build automated and computational methods to detect subtle cognitive signatures and temporal patterns that would otherwise not be recognizable.^46^, ^47^ These digital assessment protocols, including voice recordings, wearable technology inputs, passive monitoring data, and multimodal sensory measures, are already being performed in some ADRCs and are now being planned for use in most ADRCs in the near future.

ADRC and NACC infrastructure can accelerate development and evaluation of novel computational assessment approaches, leveraging uniform administration of a common protocol alongside additional ADRC‐specific content. Machine learning methods applied to digital implementations of traditional assessments, such as clock drawing and the Trail Making Test, detect cognitive impairment efficaciously.^48^, ^49^, ^50^, ^51^ Larger collections of aggregated and harmonized data are needed to assess the extent to which these findings generalize, and to facilitate comparative evaluation of novel approaches. The neuropsychological assessments conducted at ADRCs present a valuable opportunity to validate automated methods alongside traditional and modern psychometric scoring approaches.

Speech analysis represents a particularly promising area of methodological investigation. Speech production processes reflect underlying cognitive and perceptual functions.^52^ A growing body of work suggests that traditional methods of assessing speech, such as clinician judgment or manual formal speech assessment, exhibit variable reliability.^53^, ^54^, ^55^, ^56^, ^57^ Automated speech analysis methods applied to data from neuropsychological tests could enable objective, efficient, non‐invasive, and cost‐effective screening and monitoring of cognitive decline (for reviews, see Refs. [58, 59]). Speech‐derived metrics may be particularly valuable in conjunction with NACC's other harmonized cognitive data.

These machine learning approaches face several challenges before they become standardly accepted means of assessing cognition. Machine learning methods are vulnerable to overfitting, which occurs when trained models may perform exceptionally well on data used to develop the model, but fail to generalize to new, unseen data.^52^ This limitation may be particularly important with speech‐based cognitive assessments. Many published studies in this domain derive results from a single dataset, the Pitt corpus^60^ of responses to the “Cookie Theft” picture description task from the Boston Diagnostic Aphasia Examination (BDAE),^61^ and the subset of these data used in the Alzheimer's Dementia Recognition through Spontaneous Speech (ADReSS) challenge.^62^ These data do not represent the full spectrum of speech patterns that may be encountered with different populations, or in responses to other cognitive tasks.^58^, ^59^ In addition, differences in the interaction styles of test administrators across studies have been shown to influence participants’ speech patterns.^41^ This “observers’ effect”^63^ introduces unintentional and typically unaddressed variability that can undermine the reliability of speech‐based cognitive assessments. Consequently, automated speech analysis models may inadvertently capture dataset‐ or administration‐specific patterns rather than the underlying cognitive indicators they are intended to model. With the addition of speech data, the standardized infrastructure of ADRCs and NACC could present an unprecedented opportunity to validate automated cognitive assessment methods and address some of the difficulties faced by this approach to date.

There are multiple challenges to these envisioned advances. Until recently, most ADRC data collection was in American English, with some sites also collecting in Spanish and Mandarin, and Cantonese was recently added. The prior focus on American English alone limited the extent to which NACC data could inform our understanding of how AD affects linguistic capabilities in speakers of other languages, as well as in bilingual or multilingual speakers. For example, prior studies suggest that some bilingual patients with AD experience parallel deterioration in both languages, whereas others show greater impairment in the non‐dominant language over time.^64^, ^65^, ^66^, ^67^ In addition, the accuracy of linguistically based predictive models of dementia remains unknown in most languages other than English. To address these limitations, future research should implement systematic sampling of speech data from bilingual/multilingual communities and consider the use of accented speech. Cross‐linguistic studies comparing performance on cognitive tasks presented in multiple languages could reveal differential patterns of deterioration or preservation, offering more‐sensitive early markers of cognitive decline. Collaborations with international institutions would be invaluable to create diverse, multilingual datasets representative of various language families and cultural contexts. Cross‐language evaluations can be particularly tricky and require careful analytic approaches to identify aspects that function differently across different languages.^68^

## 5 THE POTENTIAL FUTURE: MEASURES OF SUBJECTIVE COGNITIVE CONCERNS

There is substantial interest in assessing participants’ subjective cognitive concerns, which can present across the lifespan and tend to increase with age, and which may be among the earliest signs of neurodegenerative disease.^68^ Older adults who report significant and persistent decline in cognitive capacity are more likely to develop dementia compared to those who do not,^68^ and in clinical settings, such reporting often prompts neuropsychological/neurological workup. Subjective reports of cognitive concerns are non‐invasive and inexpensive, which makes them attractive as accessible screening and monitoring tools in aging populations. However, the measures to query subjective cognitive concerns are highly variable with respect to key features of item format and content.^68^ The same modern psychometric approach that we have applied to harmonization of cognitive data can be applied to these measures of subjective cognitive concerns, and efforts have been made to standardize their measurement. Efforts to harmonize self‐report questionnaires^69^, ^70^ have recently resulted in a shared item bank with psychometric properties for over 600 items.^69^

Prior UDS versions did not assess subjective cognitive concerns in detail. But UDS 4 includes three items that inquire about whether the participant thinks their memory is worsening and, if so, whether this is associated with worry; the frequency of any trouble remembering things; and a rating of the participant's current memory ability as compared to 10 years ago. ADRCs interested in expanding their cognitive concerns assessment to include multiple cognitive domains beyond memory are recommended to administer the Cognitive Change Index (CCI)^71^, ^72^ or the Everyday Cognition (ECog),^73^ validated measures with self‐ and informant‐report versions. Notably, UDS 4 also includes instruments that capture contextual factors known to impact the reporting of cognitive concerns, including mood, somatic conditions, pain, medication use, sleep disorders, and psychosocial stress.^74^, ^75^, ^76^, ^77^ When possible, interpretation of cognitive concerns reporting should attempt to account for some of these variables.

An emerging direction is the incorporation of digital techniques assessing cognitive concerns repeatedly during the day as participants engage in usual daily activities.^78^, ^79^ Such methods, often referred to as ecological momentary assessments (EMAs), can account for day‐to‐day fluctuations within a real‐world environment and offer dynamic, contextually relevant data.^79^, ^80^

An important goal is to determine the individual and joint contributions of these different measurement approaches—leveraging analytic techniques such as machine learning to model the unique and shared information that these modalities provide. This could lead to the development of integrated scores that maximize the predictive accuracy of subjective cognitive concerns for AD risk and clinical progression. Traditional and novel methods for measuring cognitive concerns may provide important information relevant to therapeutic outcomes in the new era of disease‐modifying medications for AD.

## 6 THE POTENTIAL FUTURE: OTHER WAYS OF THINKING ABOUT COGNITIVE ITEMS

To date, we have focused on domain‐level scores for memory, executive functioning, and language, as well as visuospatial (when there are sufficient items for scores), in our co‐calibration work for the ADSP‐PHC. Although this is more granular than a “global cognition” construct, it is not sufficiently granular for certain research questions, and some investigators seek additional layers of granularity beyond these domain‐level constructs. For example, executive functioning items can be subdivided into subdomains such as working memory, set shifting, and abstraction, and episodic memory items can be subdivided into visual and verbal (auditory) stimuli and further subdivided into subdomains such as encoding and retention. In addition, cognitive constructs related to central auditory and visual processes, as well as motor‐ control, dual motoric, and cognitive activities, may provide a more complete view of people's cognitive aging processes. To date, although our content experts have made these distinctions, we have not incorporated these nuances into our models. The bifactor models can treat some of these elements as nuisance variables to be addressed so as to obtain psychometrically sound overall domain scores. Other choices could potentially be made when there are sufficient items available for modeling that capture some of these secondary domains.

Furthermore, items from the UDS neuropsychological battery may have utility beyond measuring cognition. In an intriguing article in this Special Issue, Gallée et al. use the UDS language items to focus on speech as a critical aspect of functional communication.^81^ Here, the same data are considered from a distinct disciplinary perspective—a speech‐language pathology perspective—as important indicators of communication with speech. Gallée et al. perform careful comparisons of speech indicators across people with early‐onset and late‐onset AD, demonstrating substantial variation in speech ability at the initial visit and visit linked in time to diagnosis.^81^

The Gallée et al. article demonstrates an important aspect of the work done by the ADSP‐PHC. With each study, the work has been extensive (as in the University of Pittsburgh ADRC paper by Choi et al.^35^), including obtaining comprehensive metadata and documenting test versions, instructions, scoring, and more. This item‐level detail is needed for our co‐calibration methods to model the constructs accurately and precisely. Concurrently, we have made available this extensive documentation, permitting others to use the same data to address scientific questions beyond those we are prioritizing for our ADSP‐PHC work.

## 7 CONCLUSIONS

We are pleased to celebrate the many accomplishments of NACC over its 25‐year history. Our work has added value to the cognitive data collected by NACC by addressing critical challenges posed by shifting batteries across UDS versions. The cognitive domain scores we have generated are useful as phenotypes for genetic and many other types of analyses. The psychometric tools we use can incorporate data beyond the UDS, which we have demonstrated adds substantial value. There are opportunities for NACC to enhance data reporting capacities for ADRCs that collect additional data beyond the UDS, and beyond the planned addition of list learning tasks. Digital data capture is an exciting technology that is amenable to modern psychometric approaches, and we look forward to its ongoing integration into ADRC activity. The quality control work we have done in generating cognitive domain scores can be leveraged by investigators addressing questions other than those we have prioritized in our work to date. NACC has facilitated an extensive array of science already. The future promises to be even more exciting.

## CONFLICT OF INTEREST STATEMENT

The authors declare no conflicts of interest. Author disclosures are available in the Supporting Information.

## Supporting information



Supporting Information
